# Overexpression of the Toll-Like Receptor (TLR) Signaling Adaptor MYD88, but Lack of Genetic Mutation, in Myelodysplastic Syndromes

**DOI:** 10.1371/journal.pone.0071120

**Published:** 2013-08-15

**Authors:** Sophie Dimicoli, Yue Wei, Carlos Bueso-Ramos, Hui Yang, Courtney DiNardo, Yu Jia, Hong Zheng, Zhihong Fang, Martin Nguyen, Sherry Pierce, Rui Chen, Hui Wang, Chenghua Wu, Guillermo Garcia-Manero

**Affiliations:** 1 Department of Leukemia, The University of Texas MD Anderson Cancer Center, Houston, Texas, United States of America; 2 Department of Hematopathology, The University of Texas MD Anderson Cancer Center, Houston, Texas, United States of America; 3 Department of Human Genetics, Baylor College of Medicine, Houston, Texas, United States of America; Cincinnati Children’s Hospital Medical Center, United States of America

## Abstract

MYD88 is a key mediator of Toll-like receptor innate immunity signaling. Oncogenically active MYD88 mutations have recently been reported in lymphoid malignancies, but has not been described in MDS. To characterize MYD88 in MDS, we sequenced the coding region of the MYD88 gene in 40 MDS patients. No MYD88 mutation was detected. We next characterized MYD88 expression in bone marrow CD34+ cells (N = 64). Increased MYD88 RNA was detected in 40% of patients. Patients with higher MYD88 expression in CD34+ cells had a tendency for shorter survival compared to the ones with lower MYD88, which was significant when controlled for IPSS and age. We then evaluated effect of MYD88 blockade in the CD34+ cells of patients with lower-risk MDS. Colony formation assays indicated that MYD88 blockade using a MYD88 inhibitor resulted in increased erythroid colony formation. MYD88 blockade also negatively regulated the secretion of interleukin-8. Treatment of MDS CD34+ cells with an IL-8 antibody also increased formation of erythroid colonies. These results indicate that MYD88 plays a role in the pathobiology of MDS and may have prognostic and therapeutic value in the management of patients with this disease.

## Introduction

The myelodysplastic syndromes (MDS) are a complex group of myeloid disorders characterized by peripheral blood cytopenias, ineffective bone marrow hematopoiesis, and increased propensity of transformation to acute myelogenous leukemia (AML) [1]. Recent use of advanced DNA sequencing technologies has allowed the identification of multiple genetic lesions in MDS [**2**]. Despite these advances, the molecular pathogenesis of MDS remains unclear. The innate immunity system is well known as a conserved host defence mechanism that detects and eliminates pathogens [3]. Activation of innate immune signaling pathways can be initiated through the stimulation of pattern-recognition receptors (PRRs), such as Toll-like receptors (TLRs) [4], with conserved molecular patterns of microorganisms. These signals are mediated via downstream signaling mediators and eventually lead to activation of key intracellular molecular effectors such as NF-kB and MAPK. The resulting immune responses, including release of inflammatory cytokines, cause elimination of pathogens. Although innate immunity responses are mediated mostly by phagocytes such as macrophages and dendritic cells, emerging evidence has suggested that innate immune signalling activation can also directly impact hematopoietic stem and early progenitor cells (HSPCs) [5], [6] and may be involved in the pathogenesis of MDS [7]. For instance, mir-145 and 146a are two microRNAs that have been shown to target the innate immune signal adaptors TIRAP and TRAF6 respectively [7]. Loss of these two microRNAs is involved in the 5q- syndrome subtype of MDS and overexpression of TRIAP and TRAF6 is associated with transformation to acute leukemia or marrow failure in a murine transplant system [8]. TRIAP and TRAF6 are both known to mediate MYD88 (Myeloid differentiation gene 88) dependent innate immune signals [4]. MYD88 mediated signaling is common to all Toll-like Receptors (TLR) except for the TLR3 pathway [9]. Of importance, oncogenically active MYD88 mutations have recently been identified as recurrent genetic lesions in chronic lymphocytic leukemia (CLL), B-cell lymphoma and Waldenström’s macroglobulinemia [10]–[12]. To evaluate if MYD88 also plays a pathological role in myeloid neoplasia, we studied MYD88 in primary samples of patients with MDS, including MYD88 mutation analysis in bone marrow mononuclear cells and the characterization of MYD88 RNA expression in bone marrow CD34+ cells and also investigated the impact of MYD88 blockade and downstream inflammatory interleukin IL-8 [13] in primary MDS CD34+ cells cultured in vitro.

## Materials and Methods

### MYD88 Gene Pyrosequencing Analysis

Pysosequencing analysis was performed in 38 patients with MDS. Exons 3 and 4 of MYD88 were amplified by polymerase chain reaction using primers listed on **Table S1**. These primers were chosen based on published data [10]–[12]. For pyrosequencing assay, the reverse primer was biotinylated. This biotinylated strand was captured on streptavidin sepharose beads (Amersham Biosciences, Uppsala, Sweden) and annealed with a sequencing primer. Pyrosequencing was performed using PSQ HS 96 Gold SNP reagents and the PSQ HS 96 pyrosequencing machine (Biotage, Uppsala, Sweden). Programmed polymorphic sites were set at specific nucleotides (see table below) to detect any mutations. Mutations were detected as abnormal program patterns (pyrosequencing peak).

### MYD88 Gene Barcode PCR-deep Sequencing Analysis

The complete coding region of MYD88 gene was amplified using ten pairs of PCR primers in 40 patients with MDS (38 described above and two additional ones). Characteristics of these patients are listed in **Table 1**. First round PCR products were then amplified in 2^nd^ round PCR using universal primers with Illumina adaptor and 40 patient-specific barcode sequences. All PCR products were then pooled together and sequenced using the Illumina HiSeq 2000 (Illumina, San Diego CA). All PCR primers are listed in **Table S2**. MYD88 sequencing dataset can be accessed at NIH Short Read Archive using ID SRP026064.

**Table 1:** Clinical Characteristics of Patients in MYD88 Mutation Analysis.

Characteristics (N = 40)		No. of Patients	%
**Age, years**			
Median	68		
Range	28–84		
**Sex**			
Male		24	60
Female		16	40
**BM blast, %**			
Median	5		
Range	0–33		
**WBC, ×10^3^/µL**			
Median	3		
Range	0.8–116.1		
**Hemoglobin, g/dL**			
Median	10		
Range	6.8–15		
**Platelet, ×10^3^/µL**			
Median	95		
Range	1–488		
**Neutrophil, ×10^3^/µL**			
Median	52		
Range	0–85		
**Prior Malignancy**			
Yes		11	27.5
No		29	72.5
**Prior Chemotherapy**			
Yes		6	15
No		34	85
**Prior Radiotherapy**			
Yes		2	5
No		38	95
**Dx**			
RARS/RCMD/RCMD-Rs		8	20
RAEB		13	32.5
CMML		3	7.5
AML/AMML		5	12.5
MPD		3	7.5
other		8	20
**IPSS**			
H		4	15
I-2		9	35
I-1		10	38
L		3	12
NA		14	
**Cytogenetics**			
Dip		23	57.5
5q-		3	7.5
other		11	27.5
IM		3	7.5

### Real-Time RT-PCR

Total cellular RNA was extracted using Trizol (Invitrogen, Carlsbad, CA) according to the manufacturer’s protocol. For each sample, 200 ng of total RNA were used for reverse transcription (RT) reactions using the High Capacity cDNA Reverse Transcription Kit (Applied Biosystems, Carlsbad, CA) following the manufacturer’s protocol. For real-time PCR, primers and probes were purchased from Applied Biosystems and analyzed using an Applied Biosystems Prism 7500 Sequence Detection System. PCR reactions were performed using 20×Assays-On-Demand Gene Expression Assay Mix and TaqMan Universal PCR Master mix according to the manufacturer’s protocol. Glyceraldehyde-3-phosphate dehydrogenase (GAPDH) was used as internal control. Quantitative values were obtained from the cycle number (C_T_ value), at which an increase in fluorescent signal associated with an exponential accumulation of PCR products was detected. The amount of target gene was normalized to the endogenous reference GAPDH to obtain the relative threshold cycle (ΔC_T_) and then related to the C_T_ of the control to obtain the relative expression level (2^–ΔΔCT^) of target gene.

### Cell Lines and Reagents

KG1 cells were obtained from ATCC (Manassas, VA) and were cultured in IMDM, 20% fatal calf serum and 1% penicillin-streptomycin. TLR-agonists MALP2 (2 µg/ml) and PAM3CSK4 (0.1 µg/ml) were purchased from Invivogen (San Diego, CA). MYD88 peptide inhibitor (Pepinh-MYD) or peptide control (5 µM) were purchased from Invivogen (San Diego, CA). IL-8 neutralization antibody (10–20 µg/ml) was purchased from ABCAM (Cambridge, MA).

### Isolation and Culture of Bone Marrow CD34+ Cells

MDS bone marrow specimens were obtained freshly from patients referred to the Department of Leukemia at MD Anderson Cancer Center following protocol PA12-0445, which is approved by Institutional Review Board (IRB) 5 of MD Anderson Cancer Center. Written informed consent from donors were obtained for use of this sample in research. Diagnosis was confirmed by a dedicated hematopathologist as soon as sample was obtained. Bone marrows from healthy individuals were obtained from AllCells (Emeryville, CA). CD34+ cells were isolated from fresh bone marrow specimens of patients with MDS using MicroBead Kit (Miltenyi, Bergisch Gladbach, Germany) following manufacturer’s instructions. Primary bone marrow CD34+ cells were cultured in IMDM (Gibco-Invitrogen), 20% BIT 9500 (bovine serum albumin, insulin, transferin) (Stem Cell Technology, Vancouver, Canada), human thrombopoietin (hTPO) 50 ng/ml (Stem Cell Technology), IL3 10 ng/ml (Stem Cell Technology), Stem cell factor (SCF) at 100 ng/ml (Stem Cell Technology), FLT3L at 100 ng/ml (Stem Cell Technology). For colony forming assays, healthy and MDS BM CD34+ cells were seeded at 1000 cells/ml and 10000 cells/ml respectively in 3.5 cm round culture dishes with methocult GF H4434 (Stem Cell Technology, Vancouver, CA). Colonies were counted after two weeks of culture.

### NF-kB Activity Evaluation by Luciferase Assay

KG1 cells were transfected with pGL4.32[*luc2P*/NF-κB-RE] (Promega, Madison, WI) using the Nucleofector Device and Nuceofector T kit (Lonza, Basel, Switzerland). These cells were pre-treated with 5 µM MYD88 peptide inhibitor or peptide control (Invivogen, San Diego, CA). pRL-TK Vectors expressing the Renilla luciferase was also cotransfected as internal control. One day after transfection, TLR2 agonist MALP2 (2 µg/ml) or PAM3CSK4 (0.1 µg/ml) (Invivogen, San Diego, CA) was added into medium. Luciferase assays were performed 20 hours later with the Dual-Luciferase Reporter Assay kit (Promega, Madison, WI) on 3010 luminometer (BD Biosciences, San Diego, CA). NF-kB activity was calculated by the ratio of firefly luminescence from the pGL4.32[*luc2P*/NF-κB-RE] reporter to the renilla luminescence from the control pRL-TK Vectors reporter.

### ELISA

IL-8 protein level in bone marrow plasma and in culture medium was analyzed using Human CXCL8/IL-8 Quantikine ELISA Kit (R&D Systems, Minneapolis, MN).

### Statistical Methods

Descriptive statistics were used for baseline characteristics. Non-parametric analyses were used; binary variables were compared using the Fisher’s exact test, the Kruskall-Wallis test was used for categorical variables, and continuous variables by the Wilcoxon rank sum test. MYD88 levels were evaluated as a continuous and categorical/dichotomous variable, using expression levels at the 25^th^, 50^th^ and 75^th^ percentiles. Associations were considered of potential interest if the *p*-value was less than 0.10. Overall survival (OS) was defined from the date of sample acquisition to date of death or date of last follow-up (censored). Kaplan-Meier analysis was used to construct OS curves; curves were compared by log-rank test. An adjusted Cox proportional hazards model was constructed using clinically important variables to examine the effect of potential confounders. We have updated this in the methods section.

## Results

### Absence of Genetic Mutations in the Coding Region of MYD88 Gene in 40 Patients with MDS

**Figure 1:**
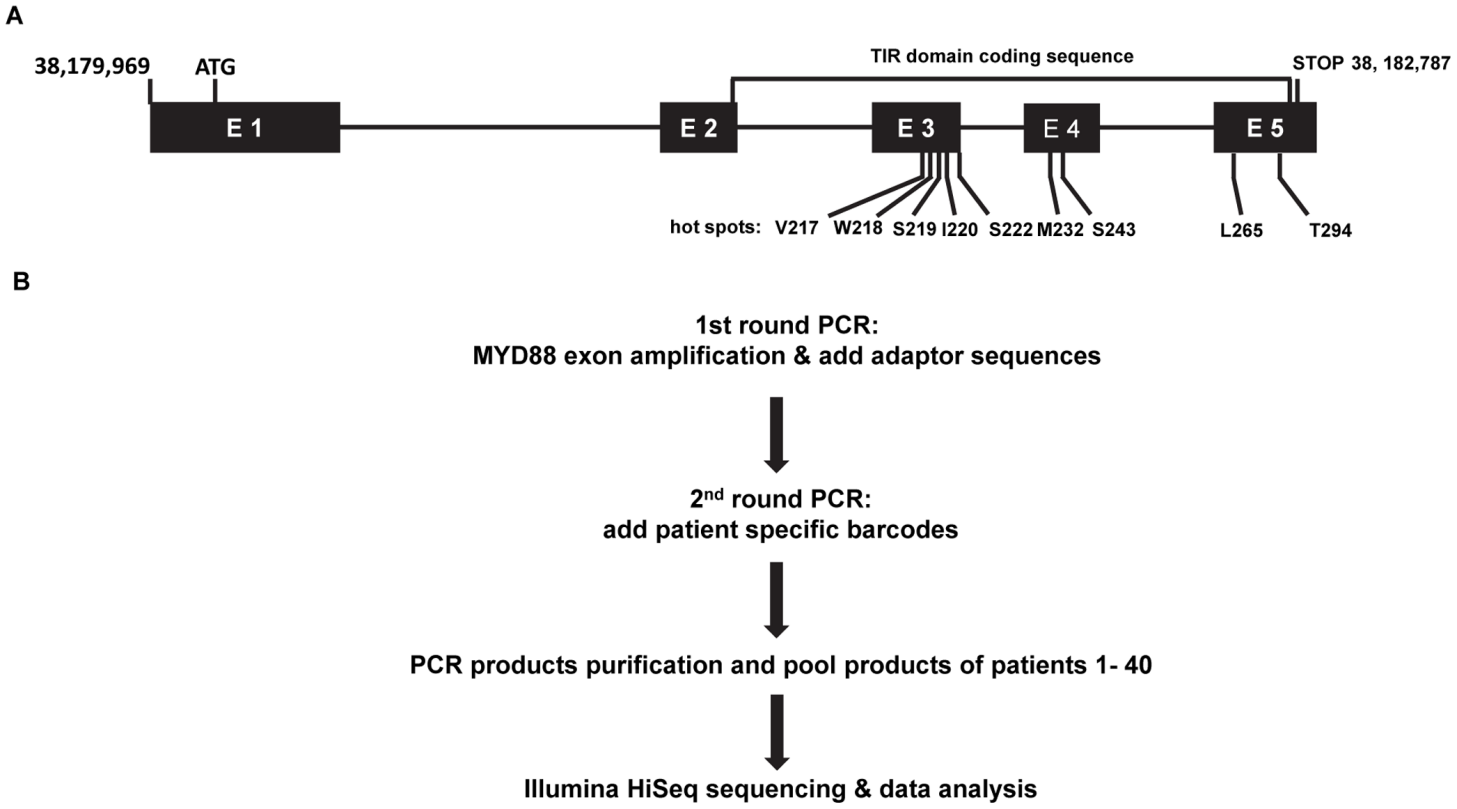
Barcode PCR-deep sequencing analysis MYD88 gene in bone marrow samples of 40 patients with MDS. (**A**) Genomic Sequence of MYD88 is analyzed in this study. Coding regions are illustrated by capitalized characters, and locations of 10 pairs of primers used in the 1^st^ round of PCR are highlighted in different colors. Sequence encoding the TIR domain is underlined. First codon (ATG) and stop-codon are squared by red lines. (**B**) Graphic illustration of the barcode PCR-deep sequencing approach used in this study.

Mutations of MYD88 have been identified in CLL [10], B cell lymphoma [11], [12] and Waldenström’s macroglobulinemia^12^, which affect several conserved amino acids in the intracellular Toll/Interleukin-1 receptor (TIR) domain (Figure 1A). We first examined these previously described “hot spots” of MYD88 in MDS using pyrosequencing approach. Pyrosequencing was performed in whole bone marrow mononuclear cells isolated from 38 patients of MDS. Characteristics of these patients are described in Table 1. Sequenced codons include: V217, W218, S219, I220, S222, M232, S243, L265, and T294. Using this technique, no mutation was detected in any of the 38 MDS samples analyzed. We then expanded the sequencing efforts by using an approach that combines bar-code PCR amplification and Illumina parallel sequencing (Figure 1B). Characteristics of these patients are listed in **Table 1**. Eleven of these patients had prior malignancies, including one with prior Brukitt’s lymphoma in remission. The entire coding region of MYD88 of 40 MDS samples, including the 38 samples used in pyrosequencing, were amplified and sequenced. No genetic mutation of MYD88 was detected in these 40 patients with this approach either.

### MYD88 is Overexpressed in Bone Marrow CD34+ Cells of MDS

**Figure 2:**
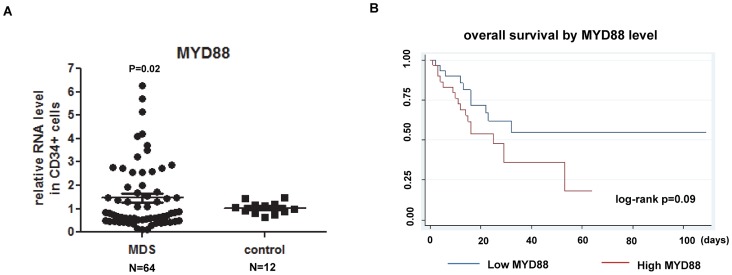
Analysis of MYD88 RNA expression in bone marrow CD34+ cells of patients with MDS. (**A**) Q-PCR results of MYD88 RNA in MDS and control CD34+ cells. (**B**) Impact of MYD88 RNA level in overall MDS patient survival.

We examined RNA expression levels of MYD88 in bone marrow CD34+ cells of patients with MDS. Analysis was performed in a cohort of 64 patients (Table 2). Characteristics of these patients are described in Table 2. Eighteen of these samples were also used for the sequencing analysis of MYD88 described earlier. We compared their MYD88 expression to 7 control normal CD34+ samples. Quantitative RT-PCR indicated that 27% of MDS patients (N = 17) had a more than 2 fold increase of MYD88 RNA, and 14% (N = 9) had a 1.3–1.9 fold increase. In total 41% (N = 26) of patients overexpress MYD88 (Figure 2A). Among these 64 patients, 35 patients (54%) were previously untreated MDS and the other 29 (45%) patients had received prior MDS treatments at the point of sample collection. Both untreated and treated patients showed increased MYD88 expression compared to controls (Figure S1A). We also analyzed MYD88 expression in these 64 patients based on their IPSS scores. MYD88 is overexpressed in patients with lower-risk (low-risk and INT-1) MDS compared to higher-risk (INT-2 and high-risk) group (p = 0.1) (Figure S1B).

**Table 2:** Clinical Characteristics of Patients in MYD88 RNA Expression Analysis.

Characteristics (N = 64)		No. of Patients	%
**Age, years**			
Median	69		
Range	33–89		
**Sex**			
Male		22	34
Female		42	66
**BM blast, %**			
Median	7		
Range	1–26		
**WBC, ×10^3^/µL**			
Median	3		
Range	0.8–67		
**Hemoglobin, g/dL**			
Median	10		
Range	7–14.2		
**Platelet, ×10^3^/µL**			
Median	67		
Range	8–488		
**Neutrophil, ×10^3^/µL**			
Median	47		
Range	0–86		
**Prior Malignancy**			
Yes		24	37
No		40	63
**Prior Chemotherapy**			
Yes		10	15
No		54	85
**Prior Radiotherapy**			
Yes		3	5
No		61	95
**Dx**			
RA/RCMD/RCMD-Rs/MDS-U		27	40
RAEB		28	43
CMML		9	14
**IPSS**			
H		9	13.8
I-2		18	27.7
I-1		26	41.5
L		11	16.9
**Cytogenetics**			
Dip, -Y		28	44.6
5/7-		15	23.1
other		18	27.7
IM		3	4.6

### MYD88 Expression and Patient Survival in MDS

We next evaluated correlations between MYD88 expression and patient characteristics in 62 of the 64 samples with available complete clinical information. Patients with higher MYD88 RNA expression in bone marrow CD34+ cells (split by median value) had a propensity for shorter survival *(p = *.09, HR 1.9, 95% CI 0.89–4.26*)* (Figure 2B). Of interest, higher MYD88 expression correlated significantly with shorter overall survival in the multivariate model adjusted for IPSS risk score and patient age (*p = *.027, HR 2.46, 95% CI 1.1–5.45). These results suggest that MYD88 expression level may hold potential prognostic value in MDS, independent of known prognostic risk factors.

### Blockade of MYD88 in MDS CD34+ Cells

**Figure 3:**
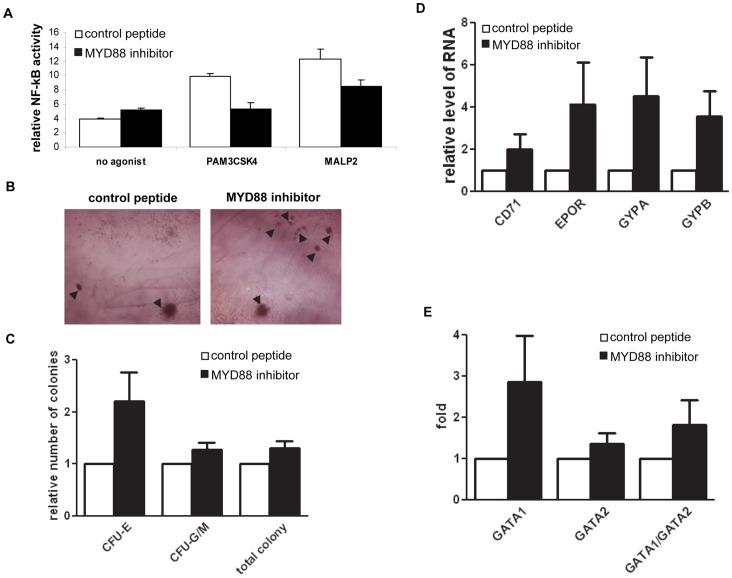
Effect of MYD88 blockade in cultured lower-risk MDS bone marrow CD34+ cells. (**A**) Analysis of the activity of the MYD88 inhibitor in KG-1 cells. Luciferase assays indicates that MYD88 inhibitor reduces the NF-kB activity that is stimulated by the TLR2 agonists PAM3CSK4 and MALP2. (**B**) Representative microphotographs of colonies derived from MDS CD34+ cells in methocult plates after two weeks of culture, treated with control peptide or MYD88 inhibitor peptide. Arrows point to CFU-E. (**C**) Colony counting after two weeks of methocult culture and treatment of MYD88 inhibitor peptide in comparison to control peptide. (**D**) Q-PCR analysis indicated elevated RNA levels of erythroid differentiation genes EPOR, CD71 and Glycophorin A and B in MYD88 inhibitor peptide treated cells in comparison to control peptide treatment. Cells were collected from methocult plates after two weeks of culture. (**E**) Q-PCR analysis of GATA1, GATA2 or GATA2/GATA1 ratio in MYD88 inhibitor peptide treated cells.

To study the role of MYD88 signaling in MDS, we used a 26-amino acid inhibitory peptide that blocks MYD88 homodimerization [14] [15]. Due to the lack of representative MDS cell lines, we first used KG1 cells, a CD34 positive cell line derived from AML [16] ^,^ [17], to examine the effect of the MYD88 peptide inhibitor. RNA levels of MYD88 are higher in KG1 cells than in 293T cell and several other leukemia cell lines such as K-562, HL-60 or U937 (data not shown). PAM3CSK4 and MALP2 are two TLR2 agonists that increase NF-kB activity in a MYD88 dependent fashion [18], [19]. These two compounds increased NF-kB promoted luciferase activity in KG1 (Figure S2). When cells were pre-incubated with the MYD88 peptide inhibitor prior to the addition of TLR2 agonists, NF-kB activation was largely reduced (Figure 3A). This result indicates that the MYD88 peptide inhibitor can block efficiently the TLR2-MYD88 dependent downstream signals.

We next applied MYD88 inhibitor on the primary bone marrow CD34+ cells isolated from patients with lower-risk MDS (IPSS low or intermediate-1) (N = 7). Patient characteristics are described in Table S3. After two weeks of culture in methylcellulose supported erythroid and myeloid differentiation medium, and in comparison to peptide control, treatment with MYD88 inhibitor resulted in increase of the number of erythroid colonies (CFU-E) in 5 of the 7 samples (Figure 3B). In average, MYD88 inhibition led to a 2 fold increase of the numbers of erythroid colonies (Figure 3C) without an obvious effect on the numbers of myeloid colonies. The impact of MYD88 blockade on differentiation of erythroid lineage in lower-risk MDS samples was further evaluated by examining the expression of genes known to be activated during erythroid differentiation. MYD88 inhibitor treatment resulted in increased expression of CD71, EPOR, GYPA and GYPB (Figure 3D) [20] ^,^ [21]. We next examined expression of GATA1 and GATA2 genes, two crucial regulators of hematopoietic differentiation, whose ratio of expression (GATA1/GATA2) has been described to positively correlate with erythroid differentiation [22] [23] [24]. We observed that while expression of both genes increased with MYD88 blockade, the increase of GATA1 was significantly higher than GATA2 (Figure 3E), leading to an increased ratio of GATA1/GATA2 (Figure 3E). These results further support the positive effect on erythroid differentiation by MYD88 blockade in low-risk MDS CD34+ cells. We also characterized effect of MYD88 blockade on the growth of MDS CD34+ cells in another three samples that were cultured in liquid hematopoietic progenitor cell expansion medium. No obvious impact on cell growth was observed (Figure S2). We next examined the effect of MYD88 inhibitor on the hematopoietic colony formation capacity of the CD34+ cells isolated from patients with clinically classified high-risk MDS (N = 3). In contrast to the observations in low-risk samples, no positive impact on erythroid colony formation was observed in any of the three high-risk samples.

### Blockade of IL-8 in MDS CD34+ Cells

**Figure 4:**
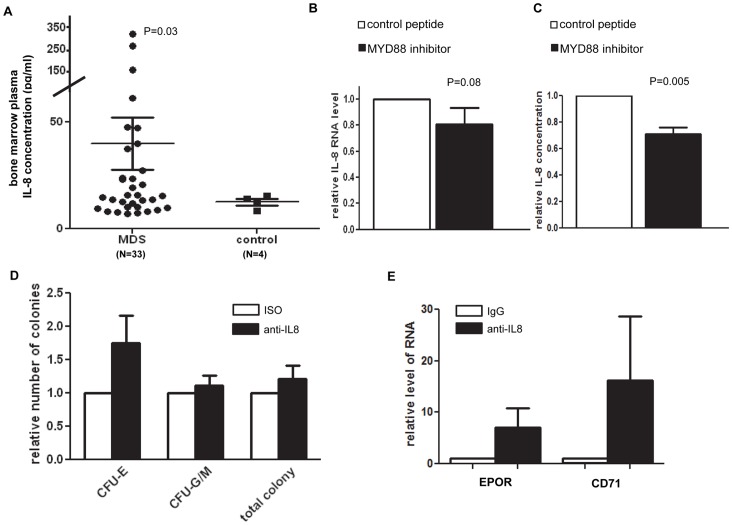
Analysis of the implication of IL-8 in MDS. (**A**) IL-8 protein concentration is elevated in bone marrow plasma samples of patients with MDS in comparison to healthy controls. (**B**). MYD88 inhibitor peptide treatment in cultured bone marrow CD34+ cells of patients with MDS leads to reduced IL-8 RNA levels. (**C**) Colony counting after two weeks of methocult culture in cells treated with IL-8 antibody or isotype control (ISO) indicates that treatment with IL-8 antibody leads to increased number of erythroid colonies in cultured CD34+ cells of patients with low-risk MDS. (**D**) Q-PCR analysis indicated elevated RNA levels of erythroid differentiation genes EPOR, and CD71 in IL-8 antibody treated cells in comparison to isotype control. Cells were collected from methocult plates after two weeks of culture.

Interleukin-8 (IL-8) is a key inflammatory cytokine dependent on MYD88 [25]. IL-8 has been shown to be one of the most significantly overexpressed cytokines in MDS BM CD34+ cells [26]. We analyzed IL-8 protein levels in the bone marrow plasma samples of patients with MDS (N = 33) and observed that IL-8 concentration was elevated in comparison to control counterparts (P = 0.03) (Figure 4A). We then examined IL-8 expression in the above described seven lower-risk MDS CD34+ samples treated with the MYD88 inhibitor. Treatment with MYD88 inhibitor resulted in a decrease of IL-8 RNA by 20% (Figure 4B). We next used ELISA to examine the IL-8 protein concentration in the supernatant of culture medium of MDS CD34+ cells (N = 4) treated with MYD88 inhibitor or control peptide. With the addition of MYD88 inhibitor, IL-8 protein concentration in culture medium was reduced by 30%, ranging between 16% to 40% in 4 samples (Figure 4C). These results suggest that MYD88 blockage negatively regulates the expression and secretion of IL-8 in MDS CD34+ cells. We then examined the effect of direct IL-8 blockade in MDS CD34+ cells by applying an IL-8 specific neutralization antibody in cells from patients with low-risk MDS (N = 5) (Table S4). A similar effect on hematopoietic differentiation of CD34+ cells was observed with the IL-8 antibody as with the MYD88 inhibitor. Colony formation assays indicated that IL-8 antibody treatment resulted in increased numbers of erythroid colonies in 4 of the 5 cases in comparison to isotype control treatment (Figure 4D). The average increase of the number of CFU-E in all 5 samples treated with IL-8 antibody was 1.7 fold in comparison to istotype control. Consistent with the positive impact on the numbers of erythroid colonies, increased CD71 and EPOR RNA expressions were also observed with IL-8 antibody treated samples (Figure 4E). No obvious increase in the RNA expression of Glycophorin A or B genes was observed after treatment with IL-8 antibody (data not shown).

## Discussion

Recently, emerging evidence has suggested a role of the TLR mediated innate immune signal in the tumorigenesis such as in gastric cancer through mechanisms independent of chronic inflammation [27]. Similar to the findings in solid tumors, deregulated innate immune signal has also been found to directly impact hematopoietic stem/early progenitor cells (HSPCs) [7] and contribute to leukomogenesis including MDS pathogenesis. [7], [28] Stimulation of TLRs is known to lead to activation of intracellular pathways such as NF-kB and p38MAPK [3], which have both been documented as important molecular signals for tumorigenesis including in MDS [29], [30]. We recently report that TLR2 [26], the histone demethylase JMJD3 and multiple innate immune genes that are regulated by JMJD3 are overexpressed in BM CD34+ cells of MDS [31]. Of importance, JMJD3 is a known transcriptional target of TLR signaling stimulated NF-kB activity [32].

As an important signal adaptor for the TLR signaling, MYD88 has been previously identified as a key contributor during the pathogenesis of solid tumors including ovarian and cutaneous malignancies [33], [34]. More recently, genetic lesions of MYD88 have recently been found to play an important role in lymphoid neoplasms, including ABC- diffuse large B cell lymphomas, central nervous system lymphomas, Waldenstrom macroglobulinemia, and also in T lymphomas [10]–[12]. Of importance, biological evidence has indicated that these recurrent genetic mutations of the MYD88 gene in lymphoma, particularly the hot-spot alteration of L265P in the TIR domain, is a gain-of-function mutation. These results implicate that MYD88 as a proto-oncogene in these lymphoid neoplasms. In this study, we were not able to detect any genetic mutation of MYD88 in bone marrow samples of MDS. In parallel to our study, the absence of L265P mutations in acute myeloid leukemia (AML), myelomonocytic leukemia, acute lymphoblastic leukemia (ALL), and multiple myeloma has been recently reported [35]. Of note, only one of the 40 patients examined for MYD88 mutation had prior history of lymphoma. This result suggests that genetic lesions of MYD88 could be specifically important for the pathogenesis of lymphoid but not myeloid neoplasms.

While no genetic lesion in the coding region of the MYD88 gene was detected in the MDS patients, we observed that RNA expression of MYD88 was abnormally increased in a substantial percentage of MDS patients in their CD34+ cells. This result is consistent with previous reports that aberrant activation of innate immune signals in MDS, including overexpression of several TLRs [36] and loss of TRAF6 targeting microRNA in the 5q- syndrome [8]. This study also suggests that MYD88 expression level was associated to the overall survival (OS) in MDS patients. The overexpression of MYD88 in MDS also suggests that although no genetic lesion has been detected in the coding regions of the gene, sequencing effort of MYD88 should also be expanded to the regulatory sequences of this gene, including promoter and potential microRNA targeting sequences that are important for its transcriptional and translational regulation. Because disease and treatment heterogeneity of patients with MDS, we performed a tentative analysis removing 21 patients with low risk morphologies (6 refractory anemia,12 refractory cytopenia multilineage dysplasia [RCMD], and 3 RCMD-RS [ring sideroblasts]). The differences in expression compared to control persisted (p = 0.1) but the impact on survival was lost (p = 0.4) (data not shown). However, most likely this effect is due to the decrease in sample size. That said, larger analysis of specific morphological subsets are indicated. Indeed one limitation of the study is the relative small sample size analysed. Another limitation is that the number of overlapping samples used for both MYD88 RNA and IL-8 Elisa analysis is very small, which makes it difficult to analyze correlation between MYD88 expression and IL-8 level. Both should be further evaluated in a larger cohort of patients in the future.

Another key finding of this study is that blockage of MYD88 and downstream IL8 in MDS CD34+ cells, particularly in CD34+ cells isolated from patients with lower-risk MDS (IPSS low-risk and intermediate-1), could positively regulate differentiation of the erythroid lineage. This observation in lower-risk samples is consistent with the observation that MYD88 expression levels tended to be higher in patients with lower-risk MDS (IPSS low-risk and intermediate-1) (Figure S1B). This effect is also consistent with our prior results of JMJD3 inhibition in MDS BM CD34+ cells [31], and that inhibition of TRAF6 in both murine and human HSPCs could prompt erythroid differentiation [8] [37]. Although the underlying molecular mechanisms are still unclear, and because anemia is one of the most common cytopenia in MDS, these observations suggest that inhibiting innate immune signals may have therapeutic role in MDS.

Overall, results of this study demonstrate that MYD88, a key innate immune signal adaptor, is potentially involved in the pathogenesis of MDS. Blockade of MYD88 mediated innate immunity signalling including IL-8 could potentially improve erythropiesis. These results further support the hypothesis that deregulation of innate immune signal contributes to the development of MDS and this is at least partially through direct impact on hematopoietic stem/progenitor cells. Further dissecting the innate immune signalling and development of pharmacological intervention of this pathway may benefit patients with MDS.

## Supporting Information

Figure S1 (A) MYD88 RNA expression level in untreated (N = 35) and treated (N = 29) MDS CD34+ cells compared to control (N = 12) CD34+ cells. (B) MYD88 RNA expression levels in BM CD34+ cells of different IPSS groups compared to control.
(PDF)

Figure S2 **Lack of effect on cell growth for MYD88 blockade in MDS CD34+ cells.**
(PDF)

Table S1 **Primers used for MYD88 Pyrosequencing.**
(PDF)

Table S2 **Primers used in bar-code sequencing of MYD88.**
(PDF)

Table S3 **Characteristics of the 7 low-risk patient MDS whose CD34+ cells treated with MYD88 inhibitor peptide.**
(PDF)

Table S4 **Characteristics of the 5 low-risk patient MDS whose CD34+ cells treated with IL-8 antibody.**
(PDF)

## References

[1] Tefferi A, Vardiman JW (2009) Myelodysplastic syndromes. N Engl J Med 361: 1872–1885. 19890130 10.1056/NEJMra0902908

[2] Bejar R, Stevenson KE, Caughey BA, Abdel-Wahab O, Steensma DP, et al (2012) Validation of a prognostic model and the impact of mutations in patients with lower-risk myelodysplastic syndromes. J Clin Oncol 30: 3376–3382. 22869879 10.1200/JCO.2011.40.7379 PMC3438234

[3] Akira S, Uematsu S, Takeuchi O (2006) Pathogen recognition and innate immunity. Cell 124: 783–801. 16497588 10.1016/j.cell.2006.02.015

[4] Kawai T, Akira S (2006) TLR signaling. Cell Death Differ 13: 816–825. 16410796 10.1038/sj.cdd.4401850

[5] Nagai Y, Garrett KP, Ohta S, Bahrun U, Kouro T, et al (2006) Toll-like receptors on hematopoietic progenitor cells stimulate innate immune system replenishment. Immunity 24: 801–812. 16782035 10.1016/j.immuni.2006.04.008 PMC1626529

[6] Esplin BL, Shimazu T, Welner RS, Garrett KP, Nie L, et al (2011) Chronic exposure to a TLR ligand injures hematopoietic stem cells. J Immunol 186: 5367–5375. 21441445 10.4049/jimmunol.1003438 PMC3086167

[7] Starczynowski DT, Karsan A (2010) Innate immune signaling in the myelodysplastic syndromes. Hematol Oncol Clin North Am 24: 343–359. 20359630 10.1016/j.hoc.2010.02.008

[8] Starczynowski DT, Kuchenbauer F, Argiropoulos B, Sung S, Morin R, et al (2010) Identification of miR-145 and miR-146a as mediators of the 5q- syndrome phenotype. Nat Med 16: 49–58. 19898489 10.1038/nm.2054

[9] Janssens S, Beyaert R (2002) A universal role for MyD88 in TLR/IL-1R-mediated signaling. Trends Biochem Sci 27: 474–482. 12217523 10.1016/s0968-0004(02)02145-x

[10] Puente XS, Pinyol M, Quesada V, Conde L, Ordonez GR, et al (2011) Whole-genome sequencing identifies recurrent mutations in chronic lymphocytic leukaemia. Nature 475: 101–105. 21642962 10.1038/nature10113 PMC3322590

[11] Ngo VN, Young RM, Schmitz R, Jhavar S, Xiao W, et al (2011) Oncogenically active MYD88 mutations in human lymphoma. Nature 470: 115–119. 21179087 10.1038/nature09671 PMC5024568

[12] Treon SP, Xu L, Yang G, Zhou Y, Liu X, et al (2012) MYD88 L265P somatic mutation in Waldenstrom’s macroglobulinemia. N Engl J Med 367: 826–833. 22931316 10.1056/NEJMoa1200710

[13] O’Connell CM, Ionova IA, Quayle AJ, Visintin A, Ingalls RR (2006) Localization of TLR2 and MyD88 to Chlamydia trachomatis inclusions. Evidence for signaling by intracellular TLR2 during infection with an obligate intracellular pathogen. J Biol Chem 281: 1652–1659. 16293622 10.1074/jbc.M510182200

[14] Loiarro M, Sette C, Gallo G, Ciacci A, Fanto N, et al (2005) Peptide-mediated interference of TIR domain dimerization in MyD88 inhibits interleukin-1-dependent activation of NF-{kappa}B. J Biol Chem 280: 15809–15814. 15755740 10.1074/jbc.C400613200

[15] Toshchakov VU, Basu S, Fenton MJ, Vogel SN (2005) Differential involvement of BB loops of toll-IL-1 resistance (TIR) domain-containing adapter proteins in TLR4- versus TLR2-mediated signal transduction. J Immunol 175: 494–500. 15972684 10.4049/jimmunol.175.1.494

[16] Ahmed N, Laverick L, Sammons J, Baumforth KR, Hassan HT (1999) Effect of all-trans retinoic acid on chemotherapy induced apoptosis and down-regulation of Bcl-2 in human myeloid leukaemia CD34 positive cells. Leuk Res 23: 741–749. 10456672 10.1016/s0145-2126(99)00084-3

[17] Koeffler HP, Golde DW (1978) Acute myelogenous leukemia: a human cell line responsive to colony-stimulating activity. Science 200: 1153–1154. 306682 10.1126/science.306682

[18] Langlet C, Springael C, Johnson J, Thomas S, Flamand V, et al (2010) PKC-alpha controls MYD88-dependent TLR/IL-1R signaling and cytokine production in mouse and human dendritic cells. Eur J Immunol 40: 505–515. 19950169 10.1002/eji.200939391

[19] Kawai T, Takeuchi O, Fujita T, Inoue J, Muhlradt PF, et al (2001) Lipopolysaccharide stimulates the MyD88-independent pathway and results in activation of IFN-regulatory factor 3 and the expression of a subset of lipopolysaccharide-inducible genes. J Immunol 167: 5887–5894. 11698465 10.4049/jimmunol.167.10.5887

[20] Filippone C, Franssila R, Kumar A, Saikko L, Kovanen PE, et al (2010) Erythroid progenitor cells expanded from peripheral blood without mobilization or preselection: molecular characteristics and functional competence. PLoS One 5: e9496. 20209110 10.1371/journal.pone.0009496 PMC2830487

[21] Sato T, Maekawa T, Watanabe S, Tsuji K, Nakahata T (2000) Erythroid progenitors differentiate and mature in response to endogenous erythropoietin. J Clin Invest 106: 263–270. 10903342 10.1172/JCI9361 PMC314307

[22] Weiss MJ, Orkin SH (1995) Transcription factor GATA-1 permits survival and maturation of erythroid precursors by preventing apoptosis. Proc Natl Acad Sci U S A 92: 9623–9627. 7568185 10.1073/pnas.92.21.9623 PMC40854

[23] Galloway JL, Wingert RA, Thisse C, Thisse B, Zon LI (2005) Loss of gata1 but not gata2 converts erythropoiesis to myelopoiesis in zebrafish embryos. Dev Cell 8: 109–116. 15621534 10.1016/j.devcel.2004.12.001

[24] Grass JA, Boyer ME, Pal S, Wu J, Weiss MJ, et al (2003) GATA-1-dependent transcriptional repression of GATA-2 via disruption of positive autoregulation and domain-wide chromatin remodeling. Proc Natl Acad Sci U S A 100: 8811–8816. 12857954 10.1073/pnas.1432147100 PMC166395

[25] Wang Q, Dziarski R, Kirschning CJ, Muzio M, Gupta D (2001) Micrococci and peptidoglycan activate TLR2–>MyD88–>IRAK–>TRAF–>NIK–>IKK–>NF-kappaB signal transduction pathway that induces transcription of interleukin-8. Infect Immun 69: 2270–2276. 11254583 10.1128/IAI.69.4.2270-2276.2001 PMC98155

[26] Wei Y, Dimicoli S, Bueso-Ramos C, Chen R, Yang H, et al.. (2013) Toll-like receptor alterations in myelodysplastic syndrome. Leukemia. 10.1038/leu.2013.180 PMC4011663 23765228

[27] Tye H, Kennedy CL, Najdovska M, McLeod L, McCormack W, et al (2012) STAT3-Driven Upregulation of TLR2 Promotes Gastric Tumorigenesis Independent of Tumor Inflammation. Cancer Cell 22: 466–478. 23079657 10.1016/j.ccr.2012.08.010

[28] Fang J, Rhyasen G, Bolanos L, Rasch C, Varney M, et al (2012) Cytotoxic effects of bortezomib in myelodysplastic syndrome/acute myeloid leukemia depend on autophagy-mediated lysosomal degradation of TRAF6 and repression of PSMA1. Blood 120: 858–867. 22685174 10.1182/blood-2012-02-407999 PMC3412348

[29] Grosjean-Raillard J, Tailler M, Ades L, Perfettini JL, Fabre C, et al (2009) ATM mediates constitutive NF-kappaB activation in high-risk myelodysplastic syndrome and acute myeloid leukemia. Oncogene 28: 1099–1109. 19079347 10.1038/onc.2008.457

[30] da Costa SV, Roela RA, Junqueira MS, Arantes C, Brentani MM (2010) The role of p38 mitogen-activated protein kinase in serum-induced leukemia inhibitory factor secretion by bone marrow stromal cells from pediatric myelodysplastic syndromes. Leuk Res 34: 507–512. 19913910 10.1016/j.leukres.2009.10.012

[31] Wei Y, Chen R, Dimicoli S, Bueso-Ramos C, Neuberg D, et al.. (2013) Global H3K4me3 genome mapping reveals alterations of innate immunity signaling and overexpression of JMJD3 in human myelodysplastic syndrome CD34+ cells. Leukemia. 10.1038/leu.2013.91 PMC4476310 23538751

[32] De Santa F, Totaro MG, Prosperini E, Notarbartolo S, Testa G, et al (2007) The histone H3 lysine-27 demethylase Jmjd3 links inflammation to inhibition of polycomb-mediated gene silencing. Cell 130: 1083–1094. 17825402 10.1016/j.cell.2007.08.019

[33] Szajnik M, Szczepanski MJ, Czystowska M, Elishaev E, Mandapathil M, et al (2009) TLR4 signaling induced by lipopolysaccharide or paclitaxel regulates tumor survival and chemoresistance in ovarian cancer. Oncogene 28: 4353–4363. 19826413 10.1038/onc.2009.289 PMC2794996

[34] Swann JB, Vesely MD, Silva A, Sharkey J, Akira S, et al (2008) Demonstration of inflammation-induced cancer and cancer immunoediting during primary tumorigenesis. Proc Natl Acad Sci U S A 105: 652–656. 18178624 10.1073/pnas.0708594105 PMC2206591

[35] Je EM, Yoo NJ, Lee SH (2012) Absence of MYD88 gene mutation in acute leukemias and multiple myelomas. Eur J Haematol 88: 273–274. 21991928 10.1111/j.1600-0609.2011.01720.x

[36] Maratheftis CI, Andreakos E, Moutsopoulos HM, Voulgarelis M (2007) Toll-like receptor-4 is up-regulated in hematopoietic progenitor cells and contributes to increased apoptosis in myelodysplastic syndromes. Clin Cancer Res 13: 1154–1160. 17317824 10.1158/1078-0432.CCR-06-2108

[37] Kumar MS, Narla A, Nonami A, Mullally A, Dimitrova N, et al (2011) Coordinate loss of a microRNA and protein-coding gene cooperate in the pathogenesis of 5q- syndrome. Blood 118: 4666–4673. 21873545 10.1182/blood-2010-12-324715 PMC3208282

