# Supplementary figures and images for: Overexpression of the Toll-Like Receptor (TLR) Signaling Adaptor MYD88, but Lack of Genetic Mutation, in Myelodysplastic Syndromes
Source: PLoS One. 2013 Aug 15;8(8):e71120. doi: 10.1371/journal.pone.0071120 (PMC3744562; doi:10.1371/journal.pone.0071120)

Figure S1

A

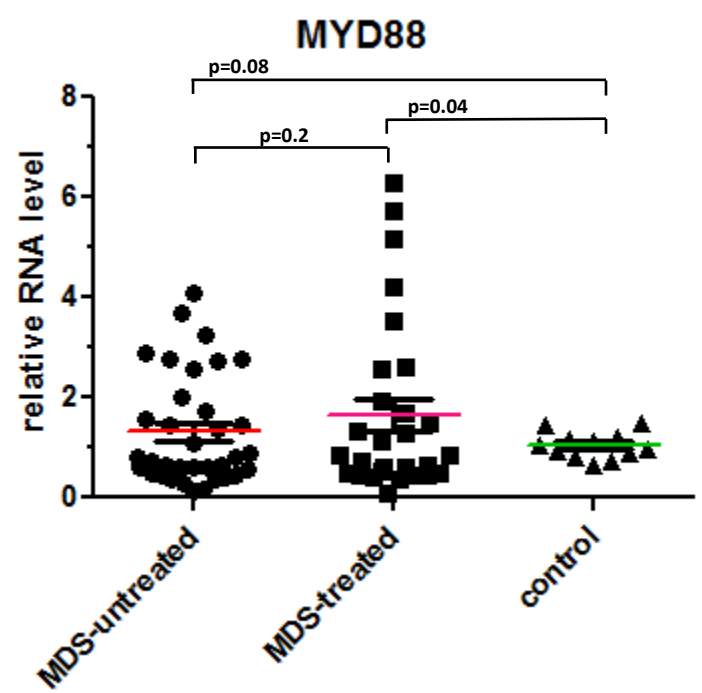

B

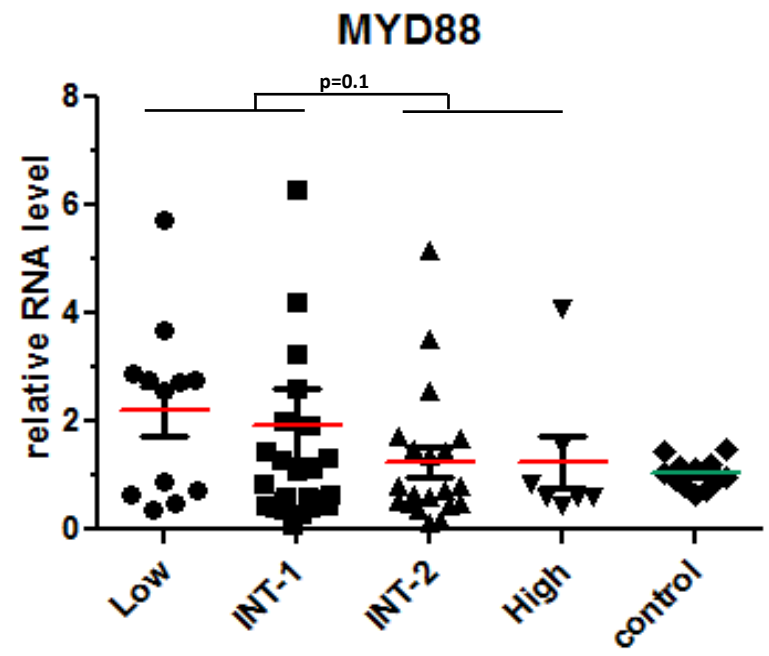

Supplement: Figure S1 — (A) MYD88 RNA expression level in untreated (N = 35) and treated (N = 29) MDS CD34+ cells compared to control (N = 12) CD34+ cells. (B) MYD88 RNA expression levels in BM CD34+ cells of different IPSS groups compared to control. (PDF) [file pone.0071120.s001.pdf]

Figure S2

A

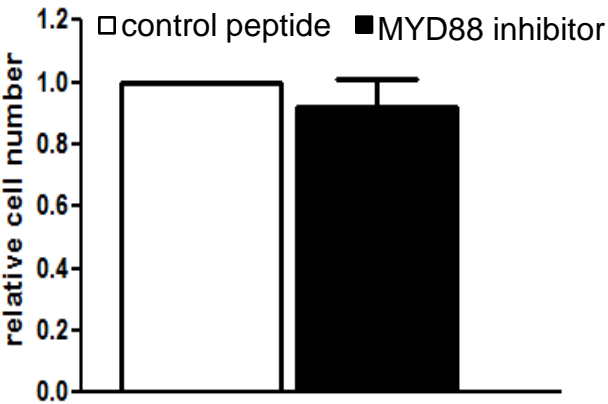

Supplement: Figure S2 — Lack of effect on cell growth for MYD88 blockade in MDS CD34+ cells. (PDF) [file pone.0071120.s002.pdf]
