# Supplementary material for: Overexpression of the Toll-Like Receptor (TLR) Signaling Adaptor MYD88, but Lack of Genetic Mutation, in Myelodysplastic Syndromes
Source: PLoS One. 2013 Aug 15;8(8):e71120. doi: 10.1371/journal.pone.0071120 (PMC3744562; doi:10.1371/journal.pone.0071120)
Supplement: Table S1 — Primers used for MYD88 Pyrosequencing. (PDF) [file pone.0071120.s003.pdf]

Table S1. Primers used for MYD88 Pyrosequencing.

**MYD88 F1**	GTGCCCATCAGAAGCGAC
**MYD88 R1**	TAGTCGCAGACAGTGATGAACC
**MYD88 Ru1**	GGGACACCGCTGATCGTTTATAGTCGCAGACAGTGATGAACC
**MYD88 S1**	TGCCCATCAGAAGCG
**MYD88 R2**	AGGGGTTGGTGTAGTCGCAGAC
**MYD88 Ru2**	GGGACACCGCTGATCGTTTAAGGGGTTGGTGTAGTCGCAGAC
**T294P F1**	ACTACACCAACCCCTGCACCA
**T294P R1**	CACACACCCAGGGCCTCA
**T294P Ru1**	GGGACACCGCTGATCGTTTACACACACCCAGGGCCTCA
**T294P S1**	GCACCAAATCTTGGTT
**M232T F1**	TGCCCACTCTCCCCTAGGT
**M232T R1**	CCAGAGCAGGGTTGAGCTTAC
**M232T Ru1**	GGGACACCGCTGATCGTTTACCAGAGCAGGGTTGAGCTTAC
**M232T S1**	TCCCCTAGGTGCCGC
**S243N F1**	GTGGTGGTTGTCTCTGATGATTAC
**S243N R1**	CACCTACACATTCCCTCATTCTC
**S243N Ru1**	GGGACACCGCTGATCGTTTACACCTACACATTCCCTCATTCTC
**S243N S1**	GTTGTCTCTGATGATTACCT
**5Mut F1**	GTGTGTCTGACCGCGATGT
**5Mut R1**	GCAGGGCTTCATGCATCC
**5Mut Ru1**	GGGACACCGCTGATCGTTTAGCAGGGCTTCATGCATCC
**5Mut S1**	CTGCCTGGCACCTGT
