# Supplementary material for: Overexpression of the Toll-Like Receptor (TLR) Signaling Adaptor MYD88, but Lack of Genetic Mutation, in Myelodysplastic Syndromes
Source: PLoS One. 2013 Aug 15;8(8):e71120. doi: 10.1371/journal.pone.0071120 (PMC3744562; doi:10.1371/journal.pone.0071120)
Supplement: Table S2 — Primers used in bar-code sequencing of MYD88. (PDF) [file pone.0071120.s004.pdf]

Table S2. Primers used in bar-code sequencing of MYD88.

1st round: (covers 6 exons of MYD88)

MYD88_Fw1: CCTACACGACGCTCTTCCGATCTGAAGCGCTGGCAGACAAT
MYD88_RV1: GTGACTGGAGTTCAGACGTGTGCTCTTCCGATCTCGCACGTTCAAGAACAGAGA

MYD88_Fw2: CCTACACGACGCTCTTCCGATCTCTCCTCCACATCCTCCCTTC
MYD88_RV2: GTGACTGGAGTTCAGACGTGTGCTCTTCCGATCTAGTTGCCGGATCTCCAAGTA

MYD88_Fw3: CCTACACGACGCTCTTCCGATCTGTACTTGGAGATCCGGCAAC
MYD88_RV3: GTGACTGGAGTTCAGACGTGTGCTCTTCCGATCTCAGGTACGAGGCCAGGAAG

MYD88_Fw4: CCTACACGACGCTCTTCCGATCTCCAGGGAGGCTGCTTTACTC
MYD88_RV4: GTGACTGGAGTTCAGACGTGTGCTCTTCCGATCTGCTTCAAACACCCATGCTCT

MYD88_Fw5: CCTACACGACGCTCTTCCGATCTTCTGACCACCACCCTTGTG
MYD88_RV5: GTGACTGGAGTTCAGACGTGTGCTCTTCCGATCTGCGGTCAGACACACACAACT

MYD88_Fw6: CCTACACGACGCTCTTCCGATCTGGCAACTGGAACAGACAAACT
MYD88_RV6: GTGACTGGAGTTCAGACGTGTGCTCTTCCGATCTGGCCTTCTAGCCAACCTCTT

MYD88_Fw7: CCTACACGACGCTCTTCCGATCTGTGTGTCTGACCGCGATGT
MYD88_RV7: GTGACTGGAGTTCAGACGTGTGCTCTTCCGATCTGCACAGCTAGGAGGAGATGC

MYD88_Fw8: CCTACACGACGCTCTTCCGATCTTGAACTAAGTTGCCACAGGAC
MYD88_RV8: GTGACTGGAGTTCAGACGTGTGCTCTTCCGATCTCCCACCTACACATTCCCTCA

MYD88_Fw9: CCTACACGACGCTCTTCCGATCTGTTGAAGACTGGGCTTGTCC
MYD88_RV9: GTGACTGGAGTTCAGACGTGTGCTCTTCCGATCTCAGTGATGAACCTCAGGATGC

MYD88_Fw10: CCTACACGACGCTCTTCCGATCTGAAGAAAGAGTTCCCCAGCA
MYD88_RV10: GTGACTGGAGTTCAGACGTGTGCTCTTCCGATCTACATGGACAGGCAGACAGAT

2nd round:
5' universal primer
AATGATACGGCGACCACCGAGATCTACACTCTTTCCCTACACGACGCTCTTCCGATCT

3' index primers

Oligo ID	Oligo sequence (5'-3') (index marked with small letters)
indexing1	CAAGCAGAAGACGGCATACGAGATcctgcgaGTGACTGGAGTTCAGACGTGTGCTCTTCCGATCT
indexing2	CAAGCAGAAGACGGCATACGAGATtgcagagGTGACTGGAGTTCAGACGTGTGCTCTTCCGATCT
indexing3	CAAGCAGAAGACGGCATACGAGATacctaggGTGACTGGAGTTCAGACGTGTGCTCTTCCGATCT
indexing4	CAAGCAGAAGACGGCATACGAGATttgatccGTGACTGGAGTTCAGACGTGTGCTCTTCCGATCT
indexing5	CAAGCAGAAGACGGCATACGAGATatcttgcGTGACTGGAGTTCAGACGTGTGCTCTTCCGATCT
indexing6	CAAGCAGAAGACGGCATACGAGATtctccatGTGACTGGAGTTCAGACGTGTGCTCTTCCGATCT
indexing7	CAAGCAGAAGACGGCATACGAGATcatcgagGTGACTGGAGTTCAGACGTGTGCTCTTCCGATCT
indexing8	CAAGCAGAAGACGGCATACGAGATttcgagcGTGACTGGAGTTCAGACGTGTGCTCTTCCGATCT
indexing9	CAAGCAGAAGACGGCATACGAGATagttggtGTGACTGGAGTTCAGACGTGTGCTCTTCCGATCT
indexing10	CAAGCAGAAGACGGCATACGAGATgtaccggGTGACTGGAGTTCAGACGTGTGCTCTTCCGATCT
indexing11	CAAGCAGAAGACGGCATACGAGATcggagttGTGACTGGAGTTCAGACGTGTGCTCTTCCGATCT
indexing12	CAAGCAGAAGACGGCATACGAGATacttcaaGTGACTGGAGTTCAGACGTGTGCTCTTCCGATCT

indexing13	CAAGCAGAAGACGGCATACGAGATtgatagtGTGACTGGAGTTCAGACGTGTGCTCTTCCGATCT
indexing14	CAAGCAGAAGACGGCATACGAGATgatccaaGTGACTGGAGTTCAGACGTGTGCTCTTCCGATCT
indexing15	CAAGCAGAAGACGGCATACGAGATcaggtcgGTGACTGGAGTTCAGACGTGTGCTCTTCCGATCT
indexing16	CAAGCAGAAGACGGCATACGAGATcgcattaGTGACTGGAGTTCAGACGTGTGCTCTTCCGATCT
indexing17	CAAGCAGAAGACGGCATACGAGATggtacctGTGACTGGAGTTCAGACGTGTGCTCTTCCGATCT
indexing18	CAAGCAGAAGACGGCATACGAGATggacgcaGTGACTGGAGTTCAGACGTGTGCTCTTCCGATCT
indexing19	CAAGCAGAAGACGGCATACGAGATgagattcGTGACTGGAGTTCAGACGTGTGCTCTTCCGATCT
indexing20	CAAGCAGAAGACGGCATACGAGATgagcatgGTGACTGGAGTTCAGACGTGTGCTCTTCCGATCT
indexing21	CAAGCAGAAGACGGCATACGAGATgttgcgtGTGACTGGAGTTCAGACGTGTGCTCTTCCGATCT
indexing22	CAAGCAGAAGACGGCATACGAGATccaatgcGTGACTGGAGTTCAGACGTGTGCTCTTCCGATCT
indexing23	CAAGCAGAAGACGGCATACGAGATcgagatcGTGACTGGAGTTCAGACGTGTGCTCTTCCGATCT
indexing24	CAAGCAGAAGACGGCATACGAGATcatattgGTGACTGGAGTTCAGACGTGTGCTCTTCCGATCT
indexing25	CAAGCAGAAGACGGCATACGAGATgacgtcaGTGACTGGAGTTCAGACGTGTGCTCTTCCGATCT
indexing26	CAAGCAGAAGACGGCATACGAGATtggcatcGTGACTGGAGTTCAGACGTGTGCTCTTCCGATCT
indexing27	CAAGCAGAAGACGGCATACGAGATgtaattgGTGACTGGAGTTCAGACGTGTGCTCTTCCGATCT
indexing28	CAAGCAGAAGACGGCATACGAGATcctatctGTGACTGGAGTTCAGACGTGTGCTCTTCCGATCT
indexing29	CAAGCAGAAGACGGCATACGAGATcaatcggGTGACTGGAGTTCAGACGTGTGCTCTTCCGATCT
indexing30	CAAGCAGAAGACGGCATACGAGATgcggcatGTGACTGGAGTTCAGACGTGTGCTCTTCCGATCT
indexing31	CAAGCAGAAGACGGCATACGAGATagtactgGTGACTGGAGTTCAGACGTGTGCTCTTCCGATCT
indexing32	CAAGCAGAAGACGGCATACGAGATtactattGTGACTGGAGTTCAGACGTGTGCTCTTCCGATCT
indexing33	CAAGCAGAAGACGGCATACGAGATccggatgGTGACTGGAGTTCAGACGTGTGCTCTTCCGATCT
indexing34	CAAGCAGAAGACGGCATACGAGATaccatgaGTGACTGGAGTTCAGACGTGTGCTCTTCCGATCT
indexing35	CAAGCAGAAGACGGCATACGAGATcggttctGTGACTGGAGTTCAGACGTGTGCTCTTCCGATCT
indexing36	CAAGCAGAAGACGGCATACGAGATtattccaGTGACTGGAGTTCAGACGTGTGCTCTTCCGATCT
indexing37	CAAGCAGAAGACGGCATACGAGATcctcctgGTGACTGGAGTTCAGACGTGTGCTCTTCCGATCT
indexing38	CAAGCAGAAGACGGCATACGAGATaggtattGTGACTGGAGTTCAGACGTGTGCTCTTCCGATCT
indexing39	CAAGCAGAAGACGGCATACGAGATgcattcgGTGACTGGAGTTCAGACGTGTGCTCTTCCGATCT
indexing40	CAAGCAGAAGACGGCATACGAGATttgcgaaGTGACTGGAGTTCAGACGTGTGCTCTTCCGATCT
