# Supplementary material for: Overexpression of the Toll-Like Receptor (TLR) Signaling Adaptor MYD88, but Lack of Genetic Mutation, in Myelodysplastic Syndromes
Source: PLoS One. 2013 Aug 15;8(8):e71120. doi: 10.1371/journal.pone.0071120 (PMC3744562; doi:10.1371/journal.pone.0071120)
Supplement: Table S3 — Characteristics of the 7 low-risk patient MDS whose CD34+ cells treated with MYD88 inhibitor peptide. (PDF) [file pone.0071120.s005.pdf]

Table S3. Characteristics of the 7 low-risk patient MDS whose CD34+ cells treated with MYD88 inhibitor peptide.

Pt	Age	Sex	GDx	Dx	IPSS	BM Blast	WBC	Hgb	Plt	Neut	Status
1	73	M	MDS	RAEB	INT-1	5	2.1	10	108	36	A
2	75	F	MDS	RCMD	Low	1	5.1	9.8	197	81	A
3	53	M	MDS	RCMD	INT-1	4	3.6	10.5	171	73	A
4	61	F	MDS	RA	INT-1	2	4	11.7	96	67	A
5	69	F	MDS	RCMD	INT-1	ND	4.1	9	200	41	A
6	70	M	MDS	5q-	INT-1	4	2.6	9.1	93	53	A
7	76	F	MDS/MPD	MDS/MPD-U	Low	1	21.8	10.9	269	95	A
