# Supplementary material for: Overexpression of the Toll-Like Receptor (TLR) Signaling Adaptor MYD88, but Lack of Genetic Mutation, in Myelodysplastic Syndromes
Source: PLoS One. 2013 Aug 15;8(8):e71120. doi: 10.1371/journal.pone.0071120 (PMC3744562; doi:10.1371/journal.pone.0071120)
Supplement: Table S4 — Characteristics of the 5 low-risk patient MDS whose CD34+ cells treated with IL-8 antibody. (PDF) [file pone.0071120.s006.pdf]

Table S4. Characteristics of the 5 low-risk patient MDS whose CD34+ cells treated with IL-8 antibody.

Pt	Age	Sex	GDx	Dx	Cyto-	IPSS	BM Blast	WBC	Hgb	Plt	Neut	Status
**1**	57	M	MDS	RCMD	Dip	INT-1	2	12.8	8.9	55	91	A
**2**	75	M	MDS/MPD	CMML	20q-	Low	3	5.6	10.9	86	56	A
**3**	66	M	MDS	RCMD-RS	Dip	Low	2	5.9	10.4	190	41	A
**4**	66	M	MDS	RCMD	abn 11q	INT-1	2	4.1	8.5	69	66	A
**5**	72	M	MDS	RAEB	Dip	Low	2	2.2	11.6	66	17	A
